# Selective Androgen Receptor Modulators (SARMs) Negatively Regulate Triple-Negative Breast Cancer Growth and Epithelial:Mesenchymal Stem Cell Signaling

**DOI:** 10.1371/journal.pone.0103202

**Published:** 2014-07-29

**Authors:** Ramesh Narayanan, Sunjoo Ahn, Misty D. Cheney, Muralimohan Yepuru, Duane D. Miller, Mitchell S. Steiner, James T. Dalton

**Affiliations:** Preclinical Research and Development, GTx, Inc., Memphis, Tennessee, United States of America; Institute of Molecular and Cell Biology, Biopolis, United States of America

## Abstract

**Introduction:**

The androgen receptor (AR) is the most highly expressed steroid receptor in breast cancer with 75–95% of estrogen receptor (ER)-positive and 40–70% of ER-negative breast cancers expressing AR. Though historically breast cancers were treated with steroidal androgens, their use fell from favor because of their virilizing side effects and the emergence of tamoxifen. Nonsteroidal, tissue selective androgen receptor modulators (SARMs) may provide a novel targeted approach to exploit the therapeutic benefits of androgen therapy in breast cancer.

**Materials and Methods:**

Since MDA-MB-453 triple-negative breast cancer cells express mutated AR, PTEN, and p53, MDA-MB-231 triple-negative breast cancer cells stably expressing wildtype AR (MDA-MB-231-AR) were used to evaluate the *in vitro* and *in vivo* anti-proliferative effects of SARMs. Microarray analysis and epithelial:mesenchymal stem cell (MSC) co-culture signaling studies were performed to understand the mechanisms of action.

**Results:**

Dihydrotestosterone and SARMs, but not bicalutamide, inhibited the proliferation of MDA-MB-231-AR. The SARMs reduced the MDA-MB-231-AR tumor growth and tumor weight by greater than 90%, compared to vehicle-treated tumors. SARM treatment inhibited the intratumoral expression of genes and pathways that promote breast cancer development through its actions on the AR. SARM treatment also inhibited the metastasis-promoting paracrine factors, IL6 and MMP13, and subsequent migration and invasion of epithelial:MSC co-cultures.

**Conclusion:**

1. AR stimulation inhibits paracrine factors that are important for MSC interactions and breast cancer invasion and metastasis. 2. SARMs may provide promise as novel targeted therapies to treat AR-positive triple-negative breast cancer.

## Introduction

Breast cancer is the most commonly diagnosed cancer in women. Over 235,000 women will be diagnosed and about 40,000 women will die from breast cancer in the United States in 2014 [1]. Breast cancer is a highly heterogeneous disease with diverse clinical (tumor size, histological subtype and grade, and lymph node status) and molecular characteristics [2]. Treatment decisions for advanced breast cancer are guided by the expression of three major therapeutic targets: estrogen receptor-α (ER), progesterone receptor (PR) and human epidermal growth factor receptor-2 (HER2) [3]. While ER-positive breast cancers are treated with ER antagonists such as fulvestrant or tamoxifen and aromatase inhibitors, HER2-positive tumors are treated with HER2 inhibitors such as Herceptin [4]. Aggressive triple negative breast cancers that do not express these three proteins and breast cancers resistant to the above mentioned treatments still await the introduction of new therapeutic options [5].

While ER, PR, and HER2 are oncogenic in breast cancer, another member of the steroid hormone receptor family, the androgen receptor (AR), has historically been considered anti-proliferative and beneficial [6],[7]. Expression of AR is prognostically favorable [6]–[8]. Until the 1970s, breast cancer was treated mostly with non-aromatizable androgens such as dihydrotestosterone or fluoxymesterone [9]–[11]. AR is the most highly expressed receptor in breast cancer with more than 75–95% of ER-positive and 40–70% of ER-negative breast cancers expressing AR [8],[12]–[15]. Evidence also suggests that the AR target gene, PSA, is a favorable prognostic marker in breast cancer [16]–[18]. A study conducted with 156 breast cancer samples to histologically determine AR and PSA expression showed that 72% of the samples expressed the two proteins with significant positive correlation between them [13]. Other studies found inverse correlation between AR expression and progression-free survival both in ER positive and triple-negative breast cancers [6],[12],[19]. One of the recent papers even recommended the use of AR as one of the three proteins to classify breast cancers [7].

Despite evidence of benefit, therapeutic efforts with androgens for breast cancer preceded knowledge regarding AR expression and the use of these agents fell from favor due to virilizing side effects, fears of aromatization to estrogen, and the advent of tamoxifen. Selective androgen receptor modulators (SARMs) are a new class of drugs under development for a variety of diseases due to their high specificity for AR, selective anabolic activity, lack of virilizing side effect, and ability to extend androgen therapy to women [20]–[22]. Enobosarm (GTx-024) is the most advanced SARM in clinical development. In multiple Phase II clinical trials, enobosarm demonstrated a significant increase in lean body mass and physical function in men and postmenopausal women without the undesirable side effects of its steroidal counterparts [23]–[25].

Organ-confined cancers acquire metastatic potential as a result of epithelial:mesenchymal stem cell (MSC) interaction. Either one of the two cell types alone lack the capability to metastasize to distant organs. Invasion of other organs arises due to paracrine factors secreted during epithelial: MSC interaction [26]. Although three factors, namely CCL5, IL6, and MMP13 are up-regulated during the interaction, *in vitro* and *in vivo* studies indicate that CCL5 is the key contributor to the metastatic characteristics of breast cancer [26]. Other studies have also identified the role of IL6 in metastasis and trastustumab-resistance [27]. These findings underscore the importance of these paracrine factors in breast cancer metastasis and abrogating these factors and subsequently epithelial:MSC interactions will prevent metastasis.

Here we provide evidences, using various preclinical models that non-aromatizable AR agonists are anti-proliferative in breast cancer cells. The growth of triple negative MDA-MB-231 breast cancer cells stably over-expressing AR (MDA-MB-231-AR) was inhibited by AR agonists, but not by antagonists or structurally similar non-binders. The growth of the above indicated tumors in nude mice was completely inhibited by SARMs, GTx-027 and GTx-024, at doses as low as 5 mg/kg/day p.o. Microarray analyses with tumors obtained from xenograft studies indicated that GTx-027 inhibited the expression of mRNA for oncogenes and induced tumor suppressor genes. GTx-027 inhibited IL6 and MMP13, but not CCL5, expression that occurs during MDA-MB-231-AR:MSC interactions. Despite the lack of effect on CCL5, GTx-027 inhibited the migration and invasion of MDA-MB-231-AR-MSC co-cultures, potentially through mechanisms independent of CCL5. These studies unambiguously demonstrate the role of AR in epithelial:MSC interaction and suggest that SARMs may represent a selective therapeutic approach to the treatment of AR-positive breast cancer.

## Materials and Methods

### Reagents

AR antibody, PG-21, was obtained from Millipore (Billerica, MA). Actin antibody was procured from Chemicon International (Temecula, CA). Platypus migration assay was obtained from Fisher Scientific (Pittsburg, PA) and transwell migration chambers were obtained from Life Technologies (Carlsbad, CA). Breast cancer cDNA array, BCRT102, was obtained from Origene (Rockville, MD). All reagents used in the study were of analytical grade.

### Cell culture

All cells, except MSCs, were obtained from ATCC (Manassas, VA) and were grown according to the instructions provided. The cell lines were authenticated by the provider and were cultured for less than 6 months after resuscitation in the laboratory. Human MSCs were obtained from Lonza (Walkersville, MD). MDA-MB-231 cells were grown in Leibovitz (L-15) medium supplemented with 10% fetal bovine serum (FBS) in a CO_2_ free incubator. Human MSCs (Lonza) were grown in MSC basal media supplemented with singlequots of growth supplement (Lonza). For transactivation studies, MDA-MB-231 cells were plated in DME+5%csFBS w/o phenol red and for growth assay the cells were plated under growth conditions.

### Transfection and transactivation assay

Plasmids and transfection assays were described earlier [28]. GRE-LUC was kindly provided by Dr. Nancy L. Weigel and Dr. Bert W O'Malley (Baylor College of Medicine, Houston, TX). Stable cell lines were generated by lentiviral infection of AR cloned into pLenti U6 Pgk-puro vector as described earlier [29],[30]. LacZ, AR, and estrogen receptor-β (ER-β) adenovirus were made at Seven Hills Bioreagents (Cincinnati, OH). For transfection, cells were plated at 90, 000 cells per well of a 24 well plate in DME+5% csFBS w/o phenol red. The cells were transfected using Lipofectamine (Invitrogen, Carlsbad, CA) with 0.25 µg GRE-LUC, 0.02 µg CMV-LUC (renilla luciferase) and 25 ng of human AR. The cells were treated 24 hrs after transfection with SARMs, DHT or an inactive isomer of SARM (SARM R-isomer) and the luciferase assay performed 48 hrs after transfection. Human AR plasmid was cloned [CR3.1 vector backbone and sequence to ensure absence of any mutation. For adenoviral infection, cells were plated in growth medium in 10 cm dishes at 4 million cells and infected with adenovirus containing the respective plasmid. Cells were harvested 24 hrs after infection and plated at 10,000 cells/well in 96 well plate in growth medium. Cells were treated for respective time points and cell viability was measured using Sulfrhodamine blue reagent (SRB). Cells left over from the 4 million cells were re-plated in 10 cm dishes and protein extracts were prepared as indicated earlier and Western blot analysis performed for AR and actin [31].

### RNA isolation and gene expression

Cells for RNA isolation were plated in 96 well plates at 10,000 cells/well. RNA was isolated using Cell-to-Ct kit (Applied Biosystems, Carlsbad, CA) and realtime PCR was performed using TaqMan primers and probes from Applied Biosystems on ABI 7900 (Applied Biosystems) and normalized to GAPDH. RNA from tumors was extracted using Qiagen RNA extraction kit (Qiagen, Valencia, CA) and the concentrations were determined using Nanodrop. RNA concentrations were normalized to the same concentration using DEPC water and the RNA was subjected to further analysis.

### Tumor xenograft experiments

All animal protocols were approved by The University of Tennessee Animal Care and Use Research Committee. Xenograft experiments were performed as previously published [28]. Briefly, a mixture of cells was suspended in 0.05 ml RPMI+10% FBS and 0.05 ml Matrigel/animal and was injected subcutaneously. Once the tumor size reached 200-300 mm^3^, the animals were randomized and treated orally with the indicated drugs formulated in Tween 80:Captex 200:water (0.8∶0.2∶9). Tumor volume and body weight were measured thrice weekly. At the end of study, animals were sacrificed, tumors excised, weighed, and stored for various analyses.

### Microarray analysis

RNA from tumors was isolated and verified qualitatively and quantitatively. Samples from each group (n = 8; 100 ng/µl; total 1000 ng) were pooled and hybridized to Affymetrix human ST2.0 gene array. Data from the array were analyzed using Ingenuity Pathway Analysis software (IPA3). The data has been deposited in “Gene Expression Omnibus” and the accession number is GSE58196.

### MDA-MB-231:MSC Co-culture experiments

MDA-MB-231-AR cells and Human MSCs were plated in 96 well plates. The MDA-MB-231-AR or MDA-MB-231-GFP cells were plated at 5000 cells/well in Leibovitz growth medium (L-15 medium) supplemented with 10% FBS and grown in 0% CO_2_. The MSC were plated at 10,000 cells/well in 100 µL of MSC basal media + MSC growth factors Singlequot. The combination of MDA-MB-231-AR or MDA-MB-231-GFP cells and MSC were plated at 5,000 and 10,000 cells/well, respectively, in 100 µL/well of MSC basal media + MSC growth factor Singlequot, and incubated at 5% CO_2_. The day after plating media was removed from each well. The cells were treated with vehicle (DMSO) or GTx-027. Three days after treatment the cells were harvested and RNA extracted using Cells to CT kit and expression of various genes measured by realtime PCR.

### Platypus and transwell migration assays

MDA-MB-231-AR and Human MSCs were plated (250,000 and 500,000 cells/well) in the top wells of 6 well plate migration chamber (Life Technologies). All the cells were plated in MSC basal media supplemented with MSC growth media singlequots. The day after plating, medium was replaced with fresh medium and treated with vehicle or GTx-027. Two days after treatment the top wells were removed and the cells that had migrated to the bottom were counted with Scepter Coulter counter.

MDA-MB-231-AR and MSC cells were plated (5,000+10,000 cells/well) in MSC basal medium supplemented with MSC growth factor singlequots in 96 well platypus cell migration plate. Stoppers were removed 24 hrs after plating and cells were treated with vehicle or 1 µM GTx-027. Cells were imaged at 0, 4, 8, 16, and 24 hrs to track the migration of cells towards the center of the plate as a measure of invasion.

### Human breast cancer array panel

Breast cancer cDNA array, BCRT102, (Data S1) was obtained from Origene (Rockville, MD). Respective TaqMan probes were mixed with TaqMan master mix added to the wells containing cDNA, and expression of various genes was evaluated using ABI 7900 realtime PCR.

### Statistical analysis

All statistical analyses were performed using JMP pro using appropriate statistical analysis. * represents P<0.05, ** represents P<0.01, *** represents P<0.001.

All *in vitro* experiments were performed in triplicate. Data are represented as mean ± S.E. Human breast cancer gene expression data and correlation were analyzed using graph pad prism.

## Results

### SARMs are AR agonists in triple-negative breast cancer cells

**Figure 1:**
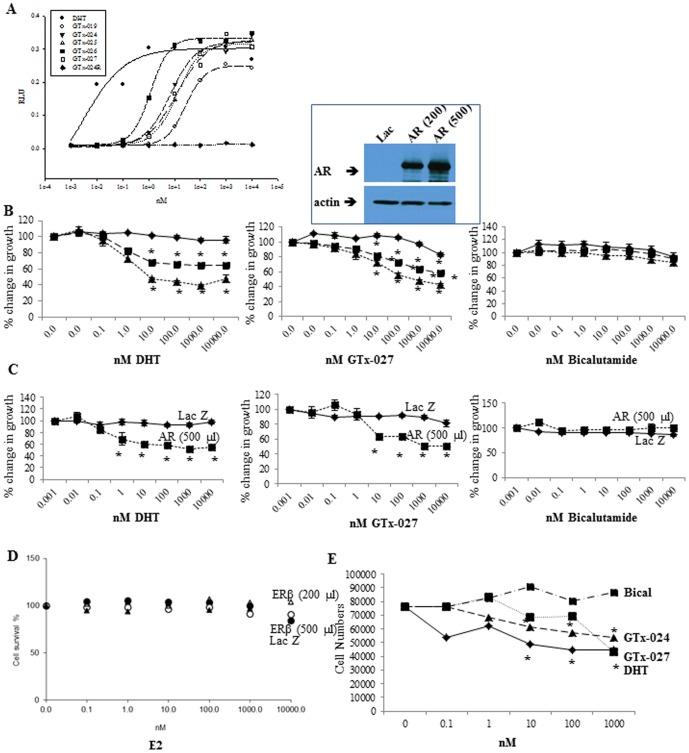
AR agonists inhibit proliferation of breast cancer cells expressing AR. **A.** AR transactivation in breast cancer cells. MDA-MB-231 cells plated in DME+5%csFBS were transfected using lipofectamine with 0.25 µg GRE-LUC, 10 ng CMV-renilla-LUC, and 25 ng CMV-hAR, treated as indicated and luciferase assay performed. **B and C.** MDA-MB-231 (B) and HCC-38 (C) breast cancer cells plated in their respective medium at 4 million cells/dish were infected with adenovirus expressing LacZ (diamond) or AR (200 µL-square; 500 µL-triangle). Twenty four hours after infection, cells were trypsinized and plated in growth medium at 10,000 cells/well in 96 well plate. Cells were treated with indicated concentrations of the drugs for 3 days. Cells were fixed and stained with sulforhodamine B (SRB) and optical density (OD) was measured at 535 nm. Inset shows AR expression in adenovirus infected MDA-MB-231 cells. **D.** Over-expressed ER-β does not inhibit MDA-MB-231 cell growth. MDA-MB-231 breast cancer cells were infected with adenovirus expressing LacZ (open triangle) or ER-β (200 µL-closed circle; 500 µL-open circle; 1000 µl-closed triangle). Cells were treated with indicated concentrations of estradiol for 3 days. Cells were fixed and stained with sulforhodamine B (SRB) and optical density (OD) was measured at 535 nm. **E.** AR agonists reduce proliferation of MDA-MB-231-AR cells. MDA-MB-231 cells stably over-expressing AR (MDA-MB-231-AR), plated in 96 well plates at 10,000 cells/well in respective medium, were treated as indicated for 6 days and the number of cells counted using coulter counter. All experiments were performed in replicates and represented as mean ± S.E.

Previous work from several groups demonstrated that local pool of cofactors and active intra-cellular signaling pathways alter the characteristics of ligands [32]–[34]. We characterized several SARMs whose structures were earlier published [35] in transient transfection and proliferation assays. Before evaluating SARMs' growth inhibitory potential in breast cancer cells, we performed AR transactivation assays to determine if the SARMs are agonists in MDA-MB-231 cells. AR transactivation assays demonstrated that SARMs are agonists in breast cancer cells (Figure 1A) and their IC_50_s and rank order were comparable to the transactivation results obtained in HEK-293 cells or COS-1 cells.

### AR agonists inhibit breast cancer cell proliferation

Triple negative breast cancer *in vitro* and *in vivo* models were developed and non-aromatizable androgens were evaluated to test the hypothesis that increasing AR function would reduce breast cancer growth. MDA-MB-231 (Figure 1B) triple negative breast cancer cells were infected with LacZ or AR adenovirus, treated with DHT, GTx-027, or bicalutamide, and cell viability was measured. Although several groups use endogenously AR-expressing MDA-MB-453 cells to evaluate androgen actions in triple negative breast cancers, MDA-MB-453 cells have AR, PTEN, and PIK3CA oncogenic mutations, HER2 over-expression and are p53 null [8]. We believe these phenotypic changes could alter the characteristics of non-steroidal AR ligands in triple-negative breast cancer cells (Figure S1). AR agonists DHT and GTx-027 reduced the proliferation of AR-expressing MDA-MB-231 (Figure 1B) cells by more than 50% compared to vehicle-treated cells. AR protein expression is shown above Figure 1B. These experiments were repeated and results were confirmed in another triple-negative breast cancer cells, HCC-38 (Figure 1C).

To understand if over-expressing other receptors in MDA-MB-231 cells elicits anti-proliferative effects in response to their respective ligand, ER-β adenovirus was prepared and used. MDA-MB-231 cells were infected with ER-β adenovirus, treated with increasing concentrations of estradiol and cell proliferation was evaluated 3 days after treatment. Though activated ER-β was shown to regulate breast cancer cell proliferation [36],[37], expression of ER-β in this cell line failed to provide any anti-proliferative effects (Figure 1D).

MDA-MB-231 cells were stably transfected with AR (MDA-MB-231-AR) and the effect of DHT and SARMs on its proliferation was evaluated and compared with bicalutamide. Corroborating the results obtained in MDA-MB-231 cells transiently expressing AR, MDA-MB-231-AR cells were also growth inhibited by DHT, GTx-024, and GTx-027, but not by bicalutamide (Figure 1E).

In addition to the above indicated ligands, proliferation of MDA-MB-231 cells in the presence of several SARMs was tested. Interestingly, all SARMs, but not antagonists, elicited anti-proliferative effects in MDA-MB-231 cells expressing AR (Table 1). AR transactivation EC_50_ results were compared with the IC50 values obtained from MDA-MB-231-AR cell growth assays (Table 1). Anti-proliferative SARMs were agonists in breast cancer cells with similar rank order for agonistic activity and anti-proliferative effects, indicating that highly potent androgens also possess robust anti-proliferative effects in MDA-MB-231-AR cells.

**Table 1:** Correlation between AR transactivation and growth inhibition of MDA-MB-231 cells with different AR ligands.

	Activity		Cell growth IC_50_ (nM)
	**EC_50_**	**IC_50_**	
**AR agonists**			
DHT	0.2		1±0.1
GTx-024	1		77±16
GTx-026	1		77±23
GTx-027	2		81±6
GTx-025	5		134±70
GTx-019	9		486±113
GTx-024R			No effect
**AR antagonists**			
Bicalutamide		22.4	No effect
CBD-I-185		6.7	No effect
CBD-IV-69		8.7	No effect

AR transactivation assay as indicated in Figure 1A was performed in MDA-MB-231 cells with the indicated ligands and represented as EC_50_. Simultaneously growth assay was performed in MDA-MB-231 cells as described in Figure 1 B and represented as IC_50_ values. All experiments were performed in replicates and represented as mean ± S.D.

### GTx-027 reduces MDA-MB-231-AR tumor growth in nude mice

**Figure 2:**
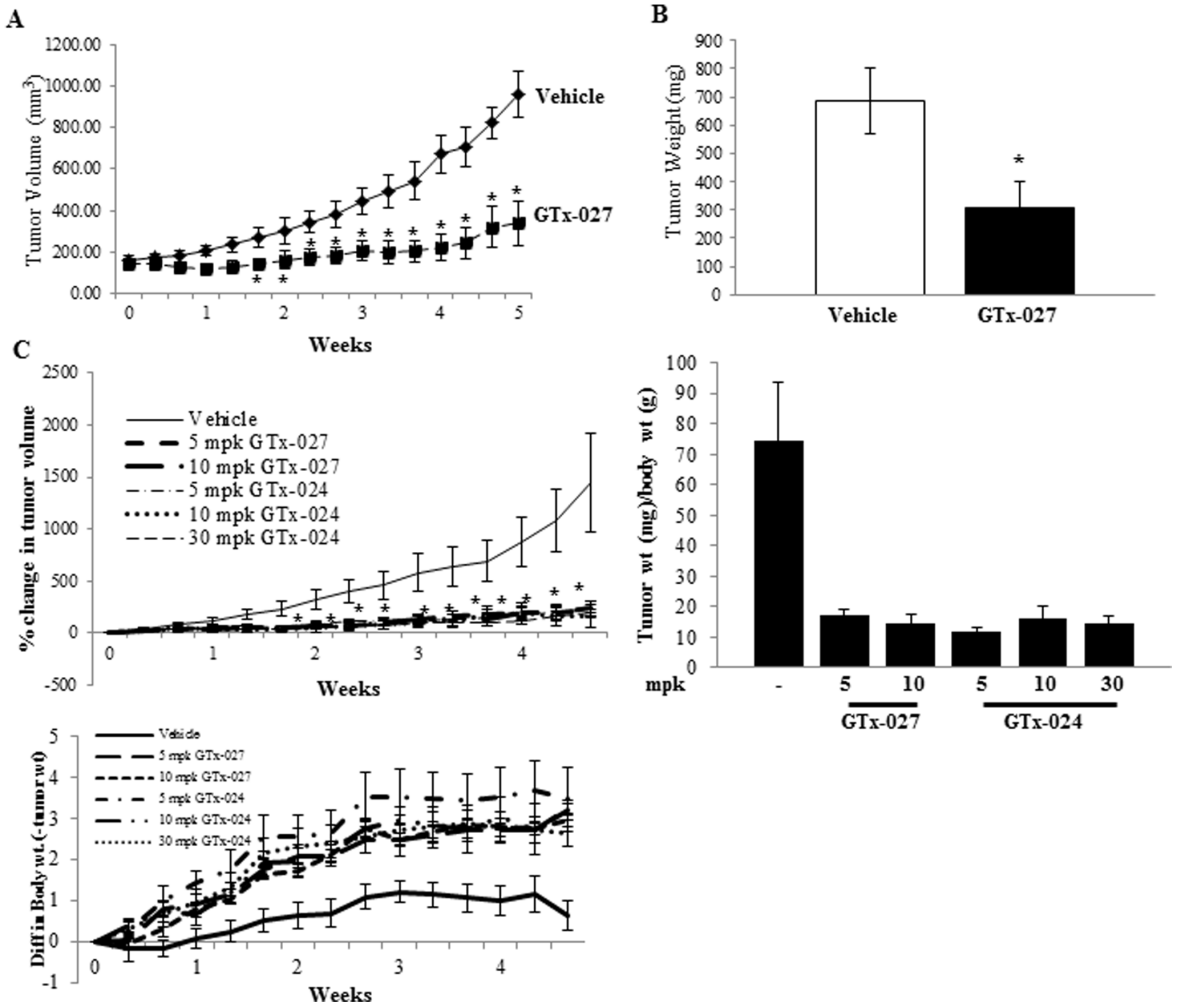
GTx SARMs inhibit triple negative breast cancer xenograft growth in nude mice. **A and B.** MDA-MB-231-AR cells (5 million cells/mouse) were mixed with matrigel and implanted subcutaneously in female nude mice (n = 8). Once tumors reached 200-300 mm^3^, animals were randomized and treated orally with vehicle or 30 mg/kg/day GTx-027. Tumor volumes (A) were measured thrice weekly. Five weeks after initiation of treatment, the animals were sacrificed, tumors weighed (B) and stored for various analyses. **C.** GTx-027 and GTx-024 inhibit tumor growth and increase body weight gain. Nude mice xenograft was performed as indicated in panel A (n = 8) with dose response of GTx-027 and GTx-024. Tumor volumes (top left panel) and body weights (bottom panel) were measured thrice weekly. At sacrifice, tumors were weighed (top right panel) and stored for further analyses. TGI-Tumor growth inhibition; *-p<0.05; ***-p<0.001. Results are represented as mean ± S.E.

To confirm the *in vitro* results in a xenograft model, MDA-MB-231-AR cells were implanted subcutaneously in female nude mice and treated orally with vehicle or 30 mg/kg/day GTx-027. While vehicle-treated tumors grew robustly from 200 mm^3^ to 1000 mm^3^ in 5 weeks, GTx-027-treated tumors grew very slowly, resulting in greater than 75% tumor growth inhibition (Figure 2A) and more than 50% tumor weight reduction (Figure 2B). Despite the high dose of GTx-027, animals did not show any toxicity, including increases in serum ALT, a classic androgenic effect in liver [38].

To ensure that GTx-027 and the clinical SARM candidate GTx-024 (Enobosarm) inhibit MDA-MB-231-AR tumor growth at lower doses, tumor bearing animals were treated orally with a dose response and tumor growth was measured. While vehicle-treated tumors grew robustly, tumor growth was completely inhibited in GTx-027- and GTx-024-treated animals (Figure 2C left panel), with tumor weight (Figure 2C right panel) reduced by greater than 90%.

One of the side effects of advanced stage cancer is cachexia, which is progressive loss of body weight and muscle mass [39]. SARMs increase lean body mass and physical function and are currently being evaluated in clinical trials as a treatment for muscle wasting associated with cancer [25],[40]. While, the vehicle-treated animals gained minimal weight during the course of the study, animals treated with GTx-024 and GTx-027 gained an average of approximately 3-5 gms weight in 5 weeks (Figure 2C bottom panel).

### Microarray analysis of MDA-MB-231-AR tumors

**Figure 3:**
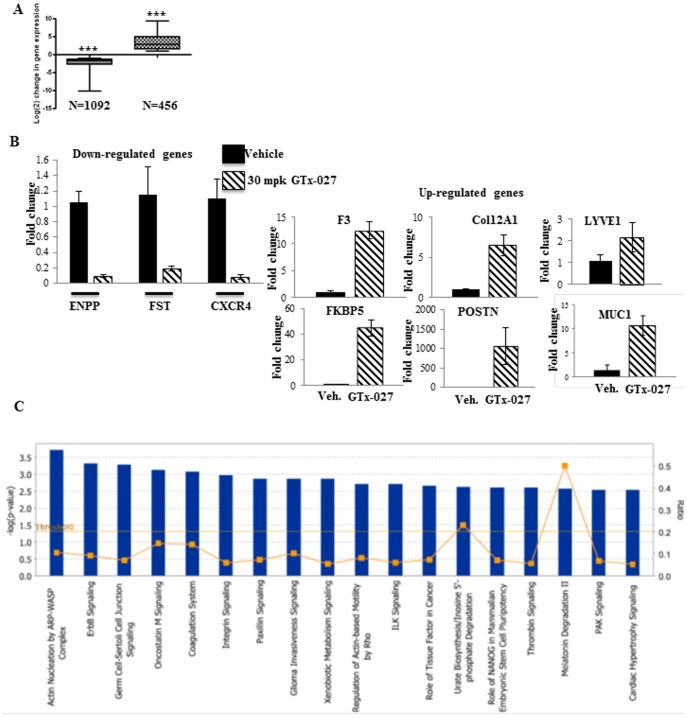
AR Agonist Negatively Regulate Cancer Genes in MDA-MB-231-AR Xenograft. **A.** Microarray analyses. RNA from tumors in panel A was isolated, pooled (n = 8/group) and subjected to microarray analysis (Affymetrix Human Gene ST2.0 array). Number of genes up- or down- regulated by GTx-027 is represented as box plot. **B.** Validation of microarray results using realtime PCR. Gene expression assays to validate the microarray results were performed in RNA from individual tumor samples (n = 8) using realtime PCR primers and probes. Expression of various genes was normalized to GAPDH. **C.** Ingenuity canonical pathway analysis. The genes that were differentially regulated by GTx-027 were analyzed using Ingenuity pathway analysis software (IPA3). The canonical pathways that are over-represented in GTx-027 treated tumors are given as bar graphs.

To evaluate the mechanism for the anti-tumor effects of SARMs in triple negative breast cancer, gene expression array studies were conducted. RNA from tumors treated with vehicle or 30 mg/kg GTx-027 were pooled and subjected to microarray analysis. Genes that were increased or decreased by 2-fold or more were considered for further analyses. Unlike in prostate cancer, where AR agonists induce more genes than they repress, in MDA-MB-231-AR tumors, GTx-027 inhibited 2.5X the number of genes (1092 *vs.* 456) than it activated (Figure 3A). Functional clustering of the genes indicated that GTx-027 modified more breast cancer genes (Table 2) than other pathway genes. Genes that regulate the function of others cancers, such as colorectal, lung, and oral, and metabolic diseases were also favorably altered by GTx-027. Breast cancer proliferative genes, such as aurora kinase, ERCC1, IGFBP3 were inhibited and growth inhibitory genes, such as NQO1, PTPRJ were activated by GTx-027 (Table 3). Many of the established androgen responsive-genes were also activated by GTx-027 (Table 4), indicating that breast cancer growth inhibitory role of GTx-027 evolved from its agonistic activity.

**Table 2:** Ingenuity pathway analysis report.

ID	Associated Network Functions			Score
1	Cell death and survival, Gastrointestinal Disease, Hepatic System Disease			37
2	Gene Expression, Cellular Movement, Cardiovascular System Development and Function			33
3	Cell Death and Survival, Cellular Movement, Cell Cycle			31
4	Cellular Movement, Cellular Development, Cellular Growth and Proliferation			29
5	Hereditary Disorder, Skeletal and Muscular Disorders, RNA Post-Transcriptional Modifications			22
**Diseases and Disorders**				
**Name**		**p-Value**	**# Molecules**	
Cancer		4.98E-08	135	
Organismal Injury and Abnormalities		7.00E-08	121	
Reproductive System Disease		7.00E-08	105	
Endocrine System Disorders		7.56E-07	33	
Infectious Disease		3.20E-06	12	
**Molecular and Cellular Functions**				
Cellular growth and functions		7.84E-10	115	
Cellular Movement		1.99E-09	74	
Cell Death and Survival		2.41E-09	110	
Cellular Development		2.19E-06	81	
Free Radical Scavenging		5.56E-05	17	
**Physiological System Development and Function**				
Cardiovascular System Development and Function		5.56E-06	39	
Organismal Development		5.56E-06	25	
Organismal Survival		8.43E-05	12	
Hematological System Development and Function		8.45E-05	19	
Hair and Skin Development and Function		9.31E-05	24	
**Top Canonical Pathways**				
Actin Nucleation by ARP-WASP Complex		1.93E-04		
ErbB Signaling		4.96E-04		
Germ Cell-Sertoli Cell Junction Signaling		5.26E-04		
Oncostatin M Signaling		7.6E-04		
Coagulation System		8.72E-04		

**Table 3:** Top disease pathway genes regulated by GTx-027 in MDA-MB-231-AR tumor xenografts.

Gene	Function	GTx-027
NQO1	Anti-proliferative, reduces oxidative stress of cells	Increased
B-Adrenoceptor2	Increases proliferation and metastasis of breast cancer, increases inflammation	Decreased
Aurora Kinase	Increases proliferation of breast cancer and aurora kinase inhibitors are effective preclinically	Decreased
BUB1 S/T kinase	Expression correlates with tumor status, node- and distant-metastasis, and histological grade in BC	Decreased
CENPE	Promotes breast cancer growth, small molecule inhibitors of CENPE inhibit BC cell growth	Decreased
EHMT2	Up-regulated in variety of cancers, including breast	Decreased
ERCC1	Expressed in 70% TNBCs and its expression leads to resistance to chemotherapy	Decreased
IGFBP3	Increases proliferative disease, higher IGFBP3 in serum correlates with higher grade disease	Decreased
ITGA2	Cancer development and metastasis	Decreased
PARP1	PARP inhibitors are currently under development for breast cancer	Decreased
POLD1	Associated with multiple cancers, including breast cancer	Decreased
PRPRJ	Tumor suppressor	Increased
SERPINE1	Tumor suppressor and inhibitor of angiogenesis, invasion and metastasis	Increased

**Table 4:** Genes involved in breast cancer growth regulated by GTx-027 in MDA-MB-231-AR tumor xenografts.

Gene	Veh	GTx-027	Fold	Function
TFPi2	876	4168	4.76	Tumor suppressor, protease inhibitor
F3	529	3672	6.94	Coagulation function
Carboxipeptidase	986	3202	3.25	Androgen-responsive gene
SNAI2/SLUG	1113	2513	2.10	Androgen-responsive gene
ASAM	581	1902	3.27	
DUSP1	413	1708	4.14	Inactivates MAPK, androgen-responsive gene
Col12a1	260	1541	5.93	
Amphiregulin	296	1322	4.47	Regulated by androgens and estrogens
Protein S	315	1163	3.69	Estrogen (down) and progestin (up) regulated gene
PDLIM1	425	1090	2.06	PR-regulated gene
FBXO32	158	1044	6.62	Androgens inhibit in muscle, promotes muscle atrophy, ubiquitin, mixed functions in cancer
RASD1	55	1044	18.62	GC-stimulated gene, Down-regulated in GC-resistant melanoma
IRS2	216	951	4.40	
FKBP51	0	541	∞	Androgen and GC stimulated
MUC1	78	704	9	Androgen and estrogen stimulated
DUSP23	125	917	7.35	Androgen-stimulated
PTGS2	59	822	14	Androgen-stimulated
RHOB	81	642	7.92	Androgen-regulated

Erbb signaling is the most affected signaling pathway by GTx-027 in MDA-MB-231-AR tumor xenografts (Figure 3C). Genes belonging to this pathway, including Amphiregulin, NCK1, NCK2, PAK, and others were differentially regulated by GTx-027. All these growth promoting genes were up-regulated by GTx-027. Hence, other powerful anti-proliferative pathways might have played a pivotal role in GTx-027's growth inhibitory effect on these tumors. In addition to Erbb signaling, other pathways such as integrin, paxillin, ILK, and PAK were also differentially regulated by GTx-027. With respect to the genes regulated in the disease and disorders category, cancer and endocrine systems disorders were the top categories influenced by GTx-027. The results from the microarray studies were deposited in “Gene Expression Omnibus” databank and the accession number is GSE58196.

A subset of GTx-027 regulated genes from the microarray was validated with individual samples using realtime PCR (Figure 3B). All genes that were taken for this validation reproduced the microarray results and the magnitude of change was much more than that observed in the array.

### GTx-027 inhibits paracrine factors secreted during epithelial:MSC interaction in MDA-MB-231-AR cells

**Figure 4:**
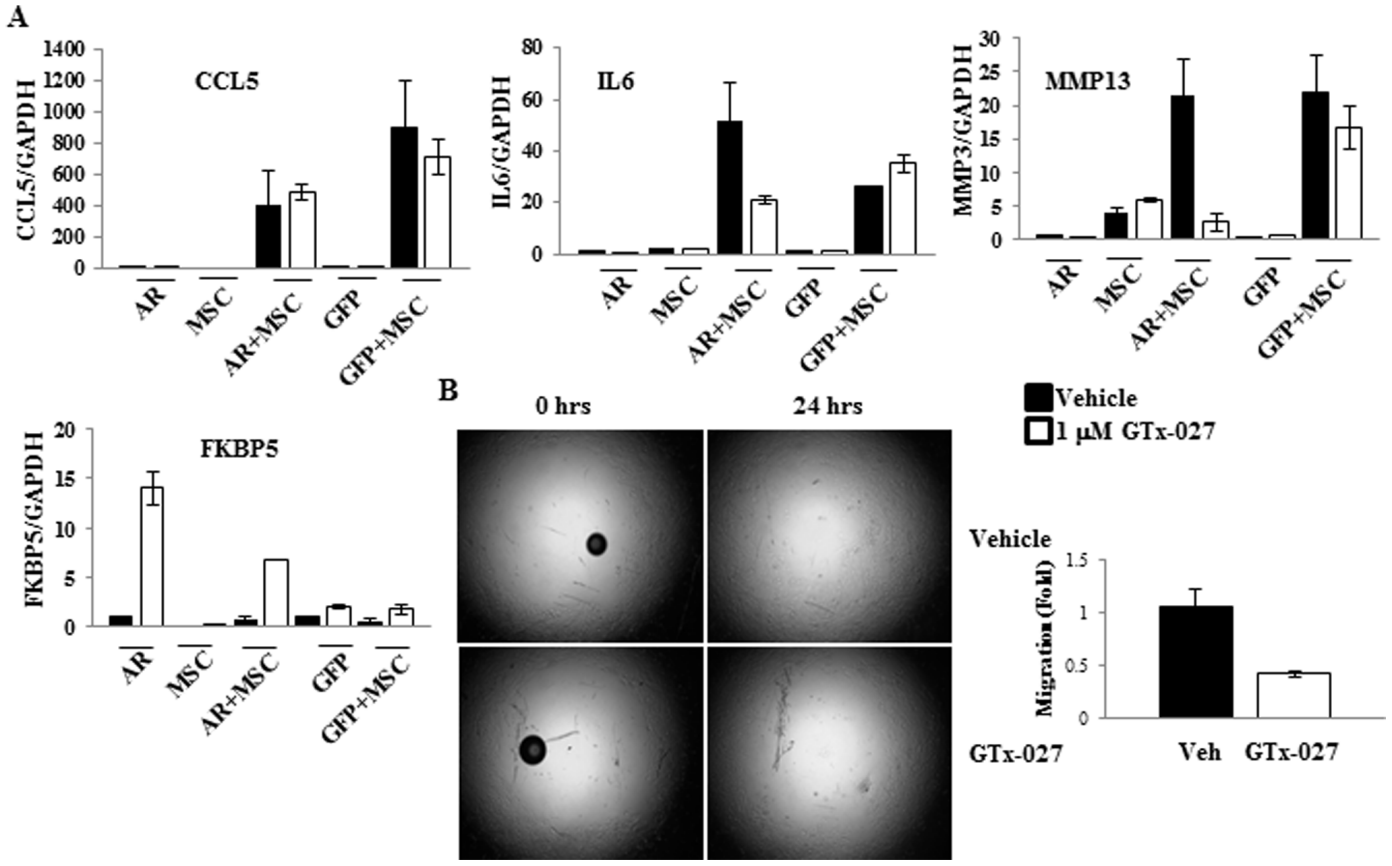
GTx-027 inhibits metastatic factors during breast cancer epithelial-mesenchymal stem cell interaction. **A.** MDA-MB-231-AR (AR) or MDA-MB-231-GFP (GFP) cells were plated alone or in combination with MSCs and treated as indicated in the figures. Three days after treatment, RNA was extracted and expression of indicated genes was measured and normalized to GAPDH using realtime PCR. **B. Left panel**: GTx-027 inhibits migration of MDA-MB-231-AR-MSCs. MDA-MB-231-AR cells were co-cultured with MSCs in platypus migration assay plate and treated with vehicle or 1 µM GTx-027. Images were acquired immediately after treatment (0 hrs) and after 24 hrs (24 hrs). **Right panel**: MDA-MB-231-AR:MSC co-cultures were plated in the upper wells of transwell migration and treated with vehicle or 1 µM GTx-027 for 3 days and the number of cells migrated from top to the bottom wells were measured twenty four hours after initiation of the experiment by staining the cells with crystal violet. Closed bars are vehicle-treated and open bars are GTx-027-treated. All experiments were performed in replicates and represented as mean ± S.E.

Epithelial:MSC interaction is a critical event preceding invasion and metastasis of breast cancer cells to distant organs [26]. Although three paracrine factors, CCL5, IL6, and MMP13, are increased during this interaction, it is well established that CCL5 is the primary mediator of the metastatic event [26]. In order to test the role of AR and its ligands during this interaction and also to understand if expression of a therapeutic target in one of the two cell types is sufficient to elicit the effect, if any, MSCs were co-cultured with MDA-MB-231-GFP or MDA-MB-231-AR cells and were treated with vehicle or GTx-027. Expression of CCL5, IL6, MMP13, and the AR target gene, FKBP5, was measured and normalized to GAPDH. As demonstrated earlier [26], CCL5, IL6, and MMP13 all increased only when MDA-MB-231 cells were co-cultured with MSCs. Interestingly, GTx-027 only inhibited the expression of IL6 and MMP13, but not the expression of CCL5, in MDA-MB-231-AR co-culture and not in MDA-MB-231-GFP co-culture (Figure 4A). As expected, GTx-027 increased FKBP5 expression in MDA-MB-231-AR cells, but not MDA-MB-231-GFP cells. GTx-027 also inhibited the effects on minimal expression of IL6 and MMP13 in MDA-MB-231-AR cells (Figure 4A), indicating that IL6 and MMP13 expression evolve from MDA-MB-231 cells and CCL5 expression from MSCs.

Platypus migration assay in MDA-MB-231-AR:MSC co-culture treated with vehicle or GTx-027 was performed to understand the effect of inhibiting two out of three paracrine factors during epithelial:MSC interaction. Surprisingly, GTx-027 inhibited the migration of cells 24 hrs after treatment initiation (Figure 4B left panel). Since AR is expressed only in MDA-MB-231 epithelial cells but not in MSCs, the regulation of migration and invasion should emanate from MDA-MB-231 cells. To confirm these results and the hypothesis, MDA-MB-231-AR:MSC co-cultures were plated in the top wells of transwell migration chambers, treated with vehicle or GTx-027, and the migration of cells from top to bottom layers was evaluated. Interestingly, GTx-027 reduced the number of cells migrated from top to the bottom (Figure 4B right panel), confirming the results obtained with platypus migration assay.

### AR function and epithelial:MSC paracrine factors inversely correlate in breast cancer samples

**Figure 5:**
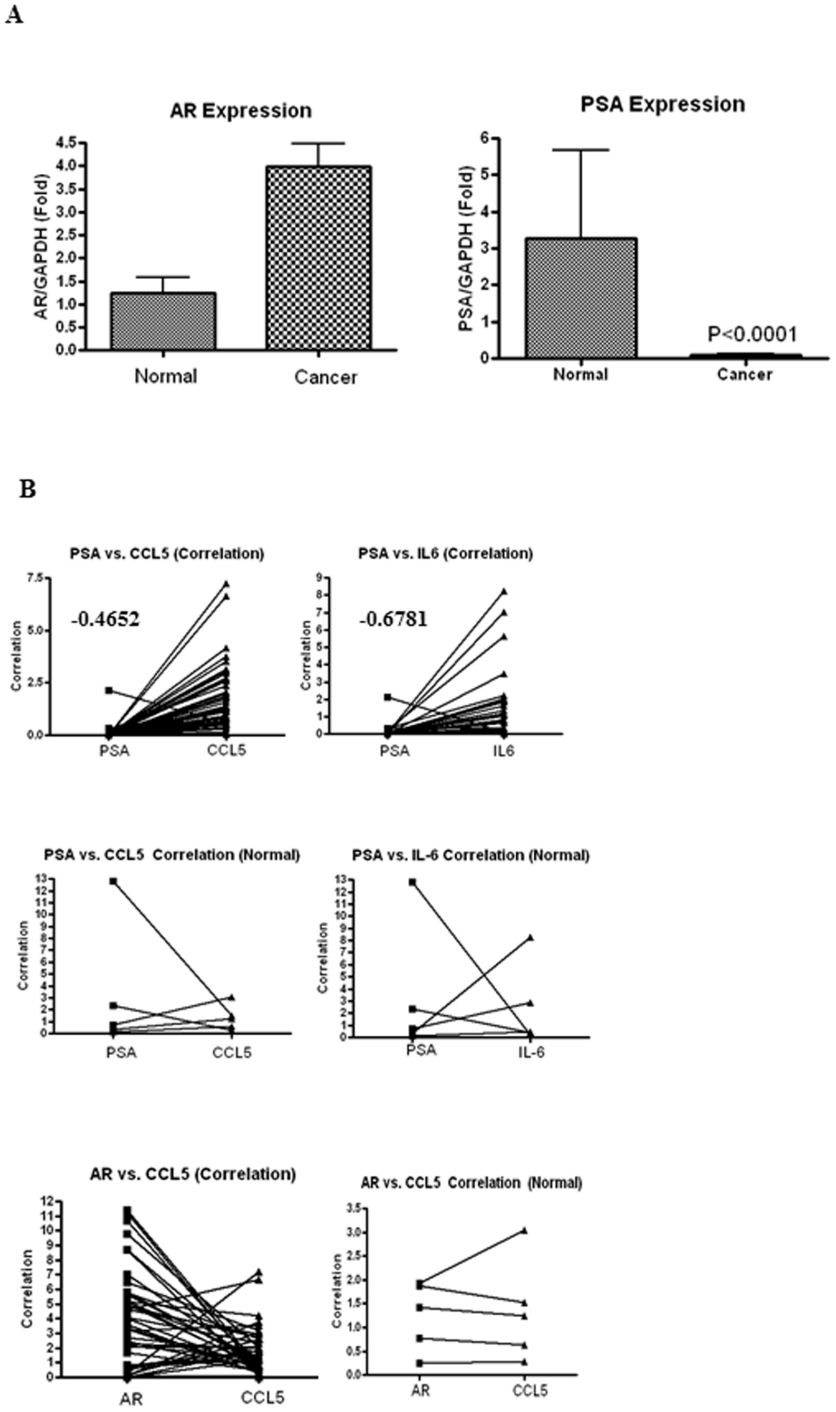
Metastatic factors inversely correlate with AR function in breast cancers. **A.** PSA gene expression is significantly reduced in breast cancer. AR and PSA gene expression were quantified by realtime PCR in cDNAs from breast cancer samples (n = 43) and normal tissues (n = 5). Gene expression was normalized to GAPDH and represented as fold difference from normal non-cancerous breast samples using ddCt method. Average of normal samples was taken for the ddCt calculation. P<0.001 for PSA. **B.** Metastatic factors inversely correlate with AR function in breast cancer. Expression of AR, PSA, CCL5, and IL6 was quantified by realtime PCR in cDNAs from 43 breast cancer samples and corresponding normal tissues. Expression of the above indicated genes was normalized to GAPDH and represented as fold difference from normal non-cancerous samples using ddCt method. Average of normal samples was taken for the ddCt calculation. Correlation between PSA or AR and CCL5 or IL6 in normal and cancerous samples was obtained and plotted as indicated in the figures. Values represented on the figures are correlation coefficient.

Although previous reports have demonstrated AR expression in breast cancer [12],[14],[19], its function in breast cancer was not quantified and compared to normal breast tissue. Breast cancer arrays containing cDNAs from 43 breast cancers and 5 normal breast tissues (Data S1) were probed for AR and its target gene, PSA. While AR expression was comparable between breast cancer and normal breast tissue, PSA expression was significantly reduced (P<0.001) in breast cancer compared to normal breast samples (Figure 5A).

In order to understand the epithelial:MSC paracrine results and their correlation with AR function in breast cancer specimen, the expression of paracrine factors were correlated with PSA. PSA expression inversely correlated with CCL5 and IL6 expression with correlation coefficients of -0.4652 and -0.6781 (Figure 5B). Impairment of AR function (Figure 5A) inversely correlates with expression of metastatic factors CCL5 and IL6 and this could be due to the repression relieved by the absence of androgens. However, this correlation did not exist in cDNAs from normal breast samples nor between AR and CCL5.

## Discussion

The role of androgens and AR in triple-negative breast cancer has been controversial for the last half a century, since the first reports of the “hyperandrogenic” theory [41],[42]. Equal evidence favors and opposes the use of androgens in breast cancer. However, most of the evidence was generated with enzymatically metabolizable androgens, such as testosterone or DHT. Studies presented herein systematically evaluate non-metabolizable AR agonists, SARMs, for the treatment of breast cancer. SARMs could evolve as a targeted therapeutic for not only ER-positive breast cancer, but also for aggressive triple negative breast cancers, for which chemotherapy is the only therapeutic option. Additionally, due to their ability to increase muscle mass and restore bone mineral density [23],[25],[43], SARMs will treat muscle wasting and osteoporosis, common side-effects in late-stage breast cancers, while reducing the aggressiveness of breast cancer.

Cancers eventually overcome the suppression elicited due to selective inhibition of a therapeutic target, by mutating or activating alternate pathways. Since AR is activated by SARMs, utilizing the AR as a therapeutic target is less likely to result in resistance. SARMs not only inhibited the growth and proliferation of breast cancer cells and tumors, but also inhibited epithelial:MSC interaction and subsequent invasion and metastasis. The results shown in Figure 4A address some fundamental questions as to which cell type secretes these paracrine factors, CCL5, IL6, and MMP13, and where the therapeutic target should be expressed to inhibit the interaction. Interestingly, the model used in this study with AR expressed only in the epithelial cells, address these questions, providing additional clarity. The results suggest that while expression of CCL5 arises from MSCs, expression of IL6 and MMP13 evolve from epithelial cells. As is evidenced by the observation that even in the absence of MSCs, epithelial cells express minimally IL6 and MMP13, which were inhibited by GTx-027 (Figure 4A). Although MSCs express higher levels of IL6 and MMP13 compared to epithelial cells, the IL6 and MMP13 produced during interaction emanate from epithelial cells.

Although these results raise the question that CCL5 might not be the only paracrine factor playing a role, it might be possible that IL6 and MMP13 are downstream targets of CCL5 and regulating these two factors might be sufficient to reduce migration and invasion. In B-cell malignancy, Waldenstrom Macroglobulinemia (WM), CCL5 modulated IL6 expression through the JAK/STAT signaling pathway [44]. While CCL5 in the absence of IL6 had no effect on migration and metastasis, CCL5 promoted migration and invasion when it had the potential to increase IL6 expression. This suggests that CCL5's function is mediated by its downstream target IL6. Similarly, another study to elucidate the mechanism for breast cancer migration and metastasis indicated that the metastasis promoted by CCL5 and CCL9 was inhibited by inhibitors of their downstream target MMP13 [45]. These results suggest that though CCL5 might be an important factor in metastasis, its effects are mediated by its downstream target IL6 and MMP13. SARMs by inhibiting the downstream targets IL6 and MMP13, abrogate migration and invasion.

Overall, these studies establish the importance of androgens and AR to treat triple-negative breast cancers.

## Supporting Information

Figure S1 **Effect of GTx-027 on proliferation of MDA-MB-453 cells.** MDA-MB-453 cells were plated in 5% charcoal stripped FBS containing L-15 medium at 10,000 cells/well in 96 well plate and incubated in 0%CO_2_ containing incubator. Medium was changed and cells were treated as indicated in the figure for 3 days. Cells were fixed, stained with SRB and the staining intensity as a measure of cell number was measured at OD 535 nm. Data are representative of n = 3 and represented as mean ± S.E.
(PPTX)

Data S1 **Microarray data table containing the genes differentially regulated by GTx-027 in MDA-MB-231-AR xenograft.**
(XLSX)

## References

[1] American Cancer Society (2014) Cancer Facts & Figures 2014. American Cancer Society.

[2] Goldhirsch A, Wood WC, Coates AS, Gelber RD, Thurlimann B, et al (2011) Strategies for subtypes—dealing with the diversity of breast cancer: highlights of the St. Gallen International Expert Consensus on the Primary Therapy of Early Breast Cancer 2011. Ann Oncol 22: 1736–1747. 21709140 10.1093/annonc/mdr304 PMC3144634

[3] Podo F, Buydens LM, Degani H, Hilhorst R, Klipp E, et al (2010) Triple-negative breast cancer: present challenges and new perspectives. Mol Oncol 4: 209–229. 20537966 10.1016/j.molonc.2010.04.006 PMC5527939

[4] Vogel VG, Costantino JP, Wickerham DL, Cronin WM, Cecchini RS, et al (2006) Effects of tamoxifen vs raloxifene on the risk of developing invasive breast cancer and other disease outcomes: the NSABP Study of Tamoxifen and Raloxifene (STAR) P-2 trial. JAMA 295: 2727–2741. 16754727 10.1001/jama.295.23.joc60074

[5] Stebbing J, Ellis P (2012) An overview of drug development for metastatic breast cancer. Br J Nurs 21: S18–22. 10.12968/bjon.2012.21.Sup4.S18 22470903

[6] Peters KM, Edwards SL, Nair SS, French JD, Bailey PJ, et al (2012) Androgen receptor expression predicts breast cancer survival: the role of genetic and epigenetic events. BMC Cancer 12: 132. 22471922 10.1186/1471-2407-12-132 PMC3349557

[7] Santagata S, Thakkar A, Ergonul A, Wang B, Woo T, et al (2014) Taxonomy of breast cancer based on normal cell phenotype predicts outcome. J Clin Invest 124: 859–870. 24463450 10.1172/JCI70941 PMC3904619

[8] Garay JP, Park BH (2012) Androgen receptor as a targeted therapy for breast cancer. Am J Cancer Res 2: 434–445. 22860233 PMC3410582

[9] Kennedy BJ (1958) Fluoxymesterone therapy in advanced breast cancer. N Engl J Med 259: 673–675. 13590423 10.1056/NEJM195810022591404

[10] Adair FE, Herrmann JB (1946) The use of testosterone propionate in the treatment of advanced carcinoma of the breast. Ann Surg 123: 1023–1035. 20987951

[11] Buchanan G, Birrell SN, Peters AA, Bianco-Miotto T, Ramsay K, et al (2005) Decreased androgen receptor levels and receptor function in breast cancer contribute to the failure of response to medroxyprogesterone acetate. Cancer Res 65: 8487–8496. 16166329 10.1158/0008-5472.CAN-04-3077

[12] Niemeier LA, Dabbs DJ, Beriwal S, Striebel JM, Bhargava R (2010) Androgen receptor in breast cancer: expression in estrogen receptor-positive tumors and in estrogen receptor-negative tumors with apocrine differentiation. Mod Pathol 23: 205–212. 19898421 10.1038/modpathol.2009.159

[13] Narita D, Raica M, Suciu C, Cimpean A, Anghel A (2006) Immunohistochemical expression of androgen receptor and prostate-specific antigen in breast cancer. Folia Histochem Cytobiol 44: 165–172. 16977795

[14] McGhan LJ, McCullough AE, Protheroe CA, Dueck AC, Lee JJ, et al (2013) Androgen Receptor-Positive Triple Negative Breast Cancer: A Unique Breast Cancer Subtype. Ann Surg Oncol 21: 361–367. 24046116 10.1245/s10434-013-3260-7

[15] Gucalp A, Tolaney S, Isakoff SJ, Ingle JN, Liu MC, et al (2013) Phase II Trial of Bicalutamide in Patients with Androgen Receptor-Positive, Estrogen Receptor-Negative Metastatic Breast Cancer. Clin Cancer Res 19: 5505–5512. 23965901 10.1158/1078-0432.CCR-12-3327 PMC4086643

[16] Narita D, Cimpean AM, Anghel A, Raica M (2006) Prostate-specific antigen value as a marker in breast cancer. Neoplasma 53: 161–167. 16575473

[17] Yu H, Diamandis EP, Levesque M, Giai M, Roagna R, et al (1996) Prostate specific antigen in breast cancer, benign breast disease and normal breast tissue. Breast Cancer Res Treat 40: 171–178. 8879683 10.1007/BF01806212

[18] Yu H, Giai M, Diamandis EP, Katsaros D, Sutherland DJ, et al (1995) Prostate-specific antigen is a new favorable prognostic indicator for women with breast cancer. Cancer Res 55: 2104–2110. 7538047

[19] Agoff SN, Swanson PE, Linden H, Hawes SE, Lawton TJ (2003) Androgen receptor expression in estrogen receptor-negative breast cancer. Immunohistochemical, clinical, and prognostic associations. Am J Clin Pathol 120: 725–731. 14608899 10.1309/42F0-0D0D-JD0J-5EDT

[20] Kearbey JD, Gao W, Narayanan R, Fisher SJ, Wu D, et al (2007) Selective Androgen Receptor Modulator (SARM) treatment prevents bone loss and reduces body fat in ovariectomized rats. Pharm Res 24: 328–335. 17063395 10.1007/s11095-006-9152-9 PMC2039878

[21] Gao W, Reiser PJ, Coss CC, Phelps MA, Kearbey JD, et al (2005) Selective androgen receptor modulator treatment improves muscle strength and body composition and prevents bone loss in orchidectomized rats. Endocrinology 146: 4887–4897. 16099859 10.1210/en.2005-0572 PMC2039881

[22] Kearbey JD, Gao W, Fisher SJ, Wu D, Miller DD, et al (2009) Effects of selective androgen receptor modulator (SARM) treatment in osteopenic female rats. Pharm Res 26: 2471–2477. 19728047 10.1007/s11095-009-9962-7

[23] Dalton JT, Barnette KG, Bohl CE, Hancock ML, Rodriguez D, et al (2011) The selective androgen receptor modulator GTx-024 (enobosarm) improves lean body mass and physical function in healthy elderly men and postmenopausal women: results of a double-blind, placebo-controlled phase II trial. J Cachexia Sarcopenia Muscle 2: 153–161. 22031847 10.1007/s13539-011-0034-6 PMC3177038

[24] Dodson S, Baracos VE, Jatoi A, Evans WJ, Cella D, et al (2011) Muscle wasting in cancer cachexia: clinical implications, diagnosis, and emerging treatment strategies. Annu Rev Med 62: 265–279. 20731602 10.1146/annurev-med-061509-131248

[25] Dobs AS, Boccia RV, Croot CC, Gabrail NY, Dalton JT, et al (2013) Effects of enobosarm on muscle wasting and physical function in patients with cancer: a double-blind, randomised controlled phase 2 trial. Lancet Oncol 14: 335–345. 23499390 10.1016/S1470-2045(13)70055-X PMC4898053

[26] Karnoub AE, Dash AB, Vo AP, Sullivan A, Brooks MW, et al (2007) Mesenchymal stem cells within tumour stroma promote breast cancer metastasis. Nature 449: 557–563. 17914389 10.1038/nature06188

[27] Korkaya H, Kim GI, Davis A, Malik F, Henry NL, et al (2012) Activation of an IL6 inflammatory loop mediates trastuzumab resistance in HER2+ breast cancer by expanding the cancer stem cell population. Mol Cell 47: 570–584. 22819326 10.1016/j.molcel.2012.06.014 PMC3432419

[28] Narayanan R, Yepuru M, Szafran AT, Szwarc M, Bohl CE, et al (2010) Discovery and mechanistic characterization of a novel selective nuclear androgen receptor exporter for the treatment of prostate cancer. Cancer Res 70: 842–851. 20068182 10.1158/0008-5472.CAN-09-3206

[29] Yang CH, Yue J, Fan M, Pfeffer LM (2010) IFN induces miR-21 through a signal transducer and activator of transcription 3-dependent pathway as a suppressive negative feedback on IFN-induced apoptosis. Cancer Res 70: 8108–8116. 20813833 10.1158/0008-5472.CAN-10-2579 PMC3014825

[30] Yepuru M, Wu Z, Kulkarni A, Yin F, Barrett CM, et al.. (2013) Steroidogenic Enzyme AKR1C3 is a Novel Androgen Receptor-Selective Coactivator That Promotes Prostate Cancer Growth. Clin Cancer Res. 10.1158/1078-0432.CCR-13-1151 23995860

[31] Narayanan R, Adigun AA, Edwards DP, Weigel NL (2005) Cyclin-dependent kinase activity is required for progesterone receptor function: novel role for cyclin A/Cdk2 as a progesterone receptor coactivator. Mol Cell Biol 25: 264–277. 15601848 10.1128/MCB.25.1.264-277.2005 PMC538783

[32] Liu Z, Auboeuf D, Wong J, Chen JD, Tsai SY, et al (2002) Coactivator/corepressor ratios modulate PR-mediated transcription by the selective receptor modulator RU486. Proc Natl Acad Sci U S A 99: 7940–7944. 12048256 10.1073/pnas.122225699 PMC122999

[33] Smith CL, O'Malley BW (2004) Coregulator function: a key to understanding tissue specificity of selective receptor modulators. Endocr Rev 25: 45–71. 14769827 10.1210/er.2003-0023

[34] Narayanan R, Coss CC, Yepuru M, Kearbey JD, Miller DD, et al (2008) Steroidal androgens and nonsteroidal, tissue-selective androgen receptor modulator, S-22, regulate androgen receptor function through distinct genomic and nongenomic signaling pathways. Mol Endocrinol 22: 2448–2465. 18801930 10.1210/me.2008-0160

[35] Chen J, Hwang DJ, Chung K, Bohl CE, Fisher SJ, et al (2005) In vitro and in vivo structure-activity relationships of novel androgen receptor ligands with multiple substituents in the B-ring. Endocrinology 146: 5444–5454. 16166218 10.1210/en.2005-0732 PMC2121105

[36] Razandi M, Pedram A, Jordan VC, Fuqua S, Levin ER (2013) Tamoxifen regulates cell fate through mitochondrial estrogen receptor beta in breast cancer. Oncogene 32: 3274–3285. 22907432 10.1038/onc.2012.335 PMC3505272

[37] Rizza P, Barone I, Zito D, Giordano F, Lanzino M, et al (2014) Estrogen receptor beta as a novel target of androgen receptor action in breast cancer cell lines. Breast Cancer Res 16: R21. 24552459 10.1186/bcr3619 PMC3978907

[38] Hild SA, Attardi BJ, Koduri S, Till BA, Reel JR (2010) Effects of synthetic androgens on liver function using the rabbit as a model. J Androl 31: 472–481. 20378929 10.2164/jandrol.109.009365 PMC2943539

[39] Fearon K, Arends J, Baracos V (2013) Understanding the mechanisms and treatment options in cancer cachexia. Nat Rev Clin Oncol 10: 90–99. 23207794 10.1038/nrclinonc.2012.209

[40] Fearon KH (2013) Selective androgen receptor modulators in cancer cachexia? Lancet Oncol 14: 271–272. 23499391 10.1016/S1470-2045(13)70068-8

[41] Grattarola R, Secreto G, Recchione C (1975) Androgens in breast cancer. III. Breast cancer recurrences years after mastectomy and increased androgenic activity. Am J Obstet Gynecol 121: 169–172. 1115123

[42] Grattarola R, Secreto G, Recchione C, Castellini W (1974) Androgens in breast cancer. II. Endometrial adenocarcinoma and breast cancer in married postmenopausal women. Am J Obstet Gynecol 118: 173–178. 4809405

[43] Furuya K, Yamamoto N, Ohyabu Y, Makino A, Morikyu T, et al (2012) The novel non-steroidal selective androgen receptor modulator S-101479 has additive effects with bisphosphonate, selective estrogen receptor modulator, and parathyroid hormone on the bones of osteoporotic female rats. Biol Pharm Bull 35: 1096–1104. 22791158 10.1248/bpb.b12-00054

[44] Elsawa SF, Novak AJ, Ziesmer SC, Almada LL, Hodge LS, et al (2011) Comprehensive analysis of tumor microenvironment cytokines in Waldenstrom macroglobulinemia identifies CCL5 as a novel modulator of IL-6 activity. Blood 118: 5540–5549. 21921047 10.1182/blood-2011-04-351742 PMC3217355

[45] Swamydas M, Ricci K, Rego SL, Dreau D (2013) Mesenchymal stem cell-derived CCL-9 and CCL-5 promote mammary tumor cell invasion and the activation of matrix metalloproteinases. Cell Adh Migr 7: 315–324. 23722213 10.4161/cam.25138 PMC3711999

