# Supplementary figures and images for: Selective Androgen Receptor Modulators (SARMs) Negatively Regulate Triple-Negative Breast Cancer Growth and Epithelial:Mesenchymal Stem Cell Signaling
Source: PLoS One. 2014 Jul 29;9(7):e103202. doi: 10.1371/journal.pone.0103202 (PMC4114483; doi:10.1371/journal.pone.0103202)

## Slide 1
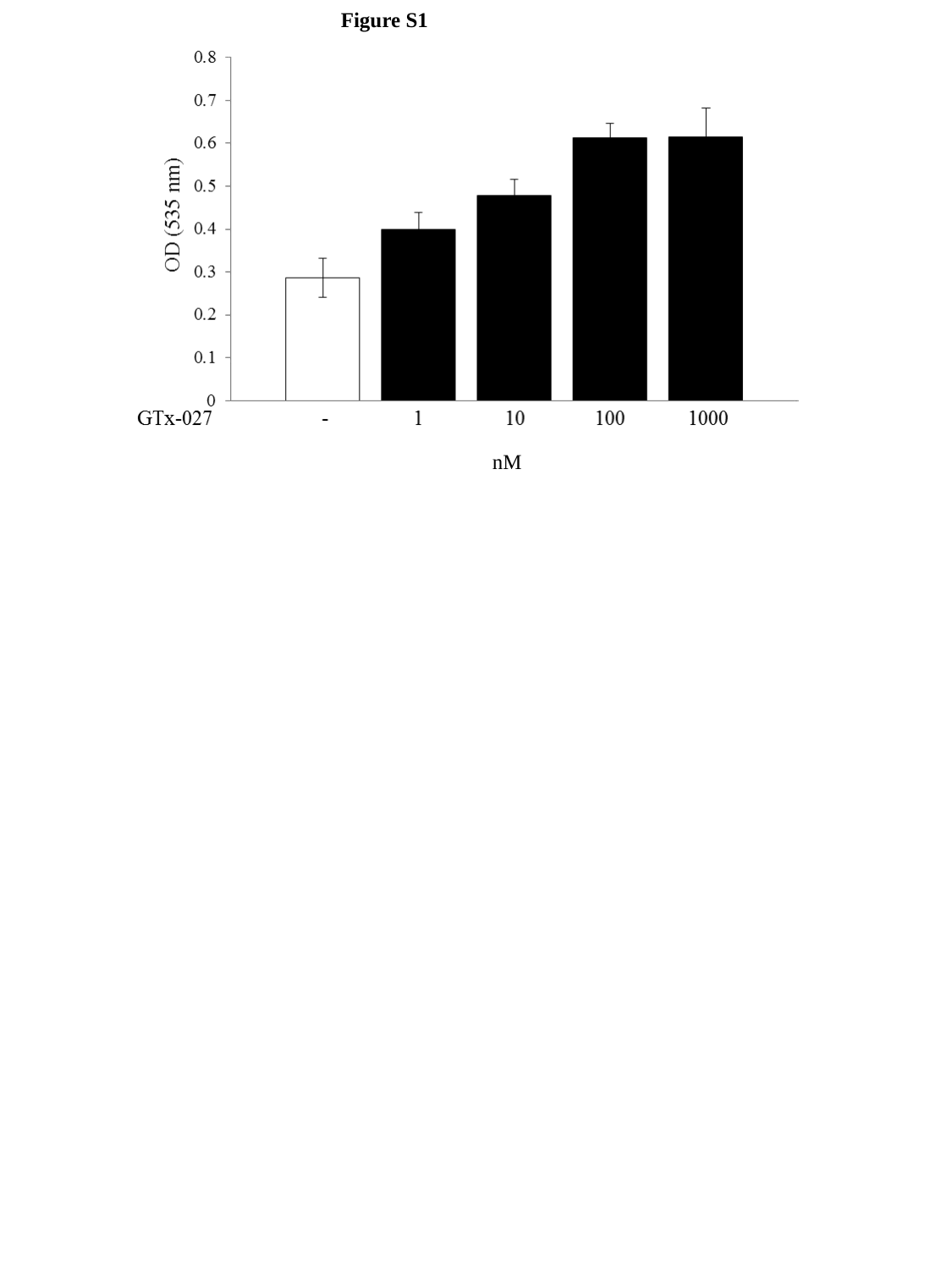

Figure S1
nM

Supplement: Figure S1 — Effect of GTx-027 on proliferation of MDA-MB-453 cells. MDA-MB-453 cells were plated in 5% charcoal stripped FBS containing L-15 medium at 10,000 cells/well in 96 well plate and incubated in 0%CO2 containing incubator. Medium was changed and cells were treated as indicated in the figure for 3 days. Cells were fixed, stained with SRB and the staining intensity as a measure of cell number was measured at OD 535 nm. Data are representative of n = 3 and represented as mean ± S.E. (PPTX) [file pone.0103202.s001.pptx]
